# Collectanea Medica

**Published:** 1812-10

**Authors:** 


### COLLECTANEA MEDICA,

CONSISTING OF

### ANECDOTES, FACTS, EXTRACTS, ILLUSTRATIONS, QUERIES, SUGGESTIONS, &c.

RELATING TO THE

*History or the Art of Medicine, and the Auxiliary Sciences.*

---

*Deductions from the Circumstances observed by Mr. Parkinson, whilst examining the several Fossils hitherto noticed.**

1st. THE water has rested for a considerable period on the general surface of the earth.

2d. The mineralized zoophytes found imbedded in different parts of the earth, and even in mountains of considerable height, have lived and died on those identical spots which, in the former world, constituted parts of the bottom of the ocean.

3d. In a previous state of this planet, many species of organized beings existed which are not known to us in a recent state; their having existed being proved only by the discovery of their fossil remains.

4th. The traces of very few of those species which now exist can be discovered in the wreck of a former world.

5th. Even in rocks of the newest formation, and in alluvial strata, which are comparatively of but modern deposition, the remains of extinct animals are as frequently to be found as in what are termed transition rocks; (those which are supposed to contain the first traces of organic remains.)

6th. There appears to have been no line of separation between the creation of species now extinct, and of those now existing; since not only the remains of extinct species, but perhaps of extinct genera, are found, with the remains of species very similar to, if not exactly agreeing with, species known in a recent state.

7th. Many of the pebbles found in gravel-pits, on the shores of rivers, and on the sea-beach, do not appear to have been bowldered down to the form in which they are now found; but that, on the contrary, their present forms are precisely those which they, at first, derived from the silicious impregnation of different animals which existed in the former ocean.

8th. Judging from the original delicacy of structure in these bodies, and from the little injury which they have sustained, it appears reasonable to suppose, that the solidi-

---

* Organic Remains of a Former World, vol. ii.

fication fication was effected, in several instances, previous to the removal of the waters from their former bed.

*Observations on the Process of Petrifaction.*

In the first volume of his valuable work on Organic Remains of a Former World, Mr. Parkinson attempted to shew that the generally-received opinion respecting the formation of petrifactions is erroneous. He supported his conjectures by a particular examination of various vegetable fossils. It has been supposed, that, in every instance of petrifaction, the lapideous is substituted for the vegetable or animal matter, as this is decomposed and removed. This removal and substitution has been also supposed to be so gradually performed, molecule by molecule, as to allow the earthy parts, whilst arranging themselves in the spaces left by the removal of the organized matter, so as to mould themselves in those spaces, as to take exactly the form of the organized part, and to imitate it precisely in every trace. In this manner the petrifaction, as it is termed, is supposed to have acquired the exact form, and most of the characteristic appearances, of the original body, without retaining any at all of its original particles. This mode of explaining the formation of petrifactions has been adopted by almost every chemist and mineralogist who has written on the subject; and has been particularly described by Kirwan, Walch, Daubenton, Fourcroy, and Haüy; the latter gentleman giving it as the explanation which is most generally admitted, although he acknowledges that it may not be free from difficulties.

Dissatisfied with this explanation, Mr. Parkinson suggested that the organized part was not removed; but that it remained in part, at least, and became the substratum of the fossil on which was deposited the lapidifying matter. He endeavored to shew that vegetable substances, in certain situations, were rendered bituminous; and were, in that state, capable of being thoroughly pervaded by water, and, of course, of being imbued with the saturated solution of any earth; and that, by the formation of minute crystallizations through the whole impregnated mass, a consolidated silicious or calcareous substance could be formed, without disturbing the existing arrangement of those parts, on which the form and general appearance of the fossil would depend. To determine how far this opinion was correct, wood petrified by flint, was subjected to simple distillation over a naked fire, when an oily sublimated film, possessing a strong empyreumatic smell, was obtained. It could not be expected, from the refractory nature of silica, that any thing more decisive could be obtained, from any experiment on opaline wood; since no agent could, perhaps, be employed for the removal of the earth, which would not at the same time entirely decompose the bituminous substratum.

But in calcareous fossil wood this objection did not exist, since, by the employment of any dilute acid, the earth might be removed, and it might be clearly ascertained whether the presumed vegetable or bituminous matter was present or not. The experiment was made, and with complete success: the carbonate of lime was removed by diluted muriatic acid, and a dark-brown, friable, but coherent, mass remained, which bore every appearance of bituminous wood, and which, when brought into contact with the flame of a candle, directly burned with a small, bright, lambent, flame, and yielded a strong bituminous odor.

From this experiment, Mr. Parkinson conceived that he was warranted in concluding that, with respect to vegetables, the process of petrifaction is not merely, as Mr. Kirwan supposes, the *substitution* of stony or metallic bodies, in the place of the organic substance which has been destroyed by putrefaction;* nor, as is taught by Fourcroy, that the petrifying matter is deposited as in a mould; the complete destruction of the vegetable matter, and the disappearance of whatever constituted its elements, taking place at the same time;† but that a part of the organic matter still remained, though somewhat changed; and that the process of petrifaction was the *impregnation* of this substance, and the filling up of the interstices with the lapidifying matter.

It still remained to determine if a correspondent organic matter existed in animal fossils. To ascertain this point, therefore, various kinds of animal fossils were subjected to a chemical examination, by which the most satisfactory proofs were obtained of the existence of this animal matter, and even of its retaining, in several instances, very much of its original form.

This circumstance was beautifully illustrated by the examination of an entrochus of the lily encrinite.‡ A similar examination of the fossil tubipore, was equally successful; the animal membrane belonging to the coral, from which the marble derived its origin, being rendered exceedingly evident. In a subsequent examination of this marble, the animal membrane was displayed, retaining the general external form, with the original color of the coral. The experiments were performed successfully with several other substances; particularly with a piece of the Derbyshire en-

* Geological Essays, p. 137.

† Systeme des Connoissances Chimiques, tome viii. p. 255.

‡ Described in Organic Remains, vol. ii. p. 166.

trochal trochal marble, the animal membrane of which was rendered perfectly distinct.

Other experiments have convinced Mr. Parkinson, that the presence of organic matter in animal fossils, of almost every kind, is sufficiently frequent to authorise, in the fullest manner, the opinion which he has advanced, respecting the principles on which the process of petrifaction is accomplished. In the vegetable fossils, it is true, that the organic matter appears to have undergone a particular change, having been previously brought to the state of bitumen; and Mr. Parkinson has strong reasons, the result of actual observation, for believing that, in the animal fossils, a correspondent change has been previously induced; and that the animal matter has suffered a conversion into adipocire.

### *Population of Great Britain.*

We had no very accurate means of estimating the population of Great Britain, previous to the late returns made to Parliament in the years 1802 and 1811, so that the estimates made are probably incorrect. But there can be no doubt that the population of the island has been constantly on the increase, at least since the revolution, and, probably, for a long period before it. In the year 1755, Brakenridge reckoned the whole inhabitants of England at 6 millions,* while Forster reckoned them at 7½ millions.† The last, notwithstanding the plausible arguments of Brakenridge to the contrary, was probably nearest the truth. At present the population is above 10 millions. It has increased (at least the population of Great Britain,) 1,600,000 within the last ten years. And, supposing the whole population of Great Britain to be 12 millions, which is not very far from the truth, then it follows that, at the present rate of increase, the number of inhabitants doubles in seventy years. This is a degree of rapidity which no body was aware of before the late Parliamentary returns. It ought to check that spirit of despondency which some of our political writers are apt to indulge. Great Britain is at present in such a state that, with frugality and wisdom, we could continue a contest with all Europe for fifty years to come, without any material injury to the nation. Indeed, in some points of view, the present state of exclusion from the continent may be considered as advantageous. To those

* Phil. Trans. 1755. Vol. xlix. p. 268.

† Phil. Trans. 1757. Vol. l. p. 457.

politicians, indeed, who consider money as the only article of value in a nation, nothing can be said: but, to those wiser men who regard the moral principles and spirit of a nation as of more consequence than wealth, it may afford consolation to consider that, by this exclusion, our young men of fortune are prevented from imbibing the profligate principles which are common on the continent, and which they were imbibing with such eagerness, that a few years of peace would have brought Great Britain to the state of profligacy so glaring in France, or to the state of imbecility and meanness so deplorable among the higher ranks in Spain.

Between the years 1740 and 1750, the number of inhabitants in Bristol, as ascertained with a good deal of accuracy by Mr. Browning, was 43,692.* Since that period the population has increased considerably, but not in the same proportion as that of several other towns in Great Britain. Various causes have checked the rapid increase of trade in Bristol; the chief probably is, that the trade of the town has got into the hands of a few rich merchants, who do not encourage the adventurous spirit of young men destitute of fortune, upon whom the increase of most places in trade and wealth, and consequently in population, depends. Bristol was formerly the second town in Great Britain in point of population, now it is exceeded by nine or ten. Two of the most remarkable examples of increase of population are Liverpool and Sheffield. Liverpool, in Queen Elizabeth's time, was hardly inhabited at all; at present its population is not much short of 100,000. Sheffield, in Queen Elizabeth's time, did not contain more than 2000 inhabitants: its present population exceeds 53,000. In the year 1773, the number of inhabitants in Manchester, from an actual survey, was 27,246. By the returns made to Parliament in 1811, they amount to about 100,000; and, if we include Salford, which, in reality constitutes a part of the town, as much as Southwark does of London, the present population of Manchester is about 128,000.

Chester is one of the oldest towns in Great Britain. From the name, and from many other circumstances, there can be no doubt that it was a Roman station. It has long been resorted to as an agreeable retirement for persons of small incomes, both on account of the comparative cheapness of the place, and the agreeableness of the country.

* Phil. Trans. 1753. Vol. xlviii. p. 217.

There is another inducement of no less importance, of which, perhaps, people in general are not aware. From a comparison of the bills of mortality at Chester with those in other places, Dr. Haygarth has shown that it is one of the healthiest spots in Great Britain.*

Dr. White, by comparing the births in York, between 1728 and 1735, with those between 1770 and 1776, has shown that the city has increased prodigiously in healthiness. At the former period the burials exceeded the births by 98 annually, the average number of burials being 498. At the latter period the births exceeded the burials by $21\frac{1}{7}$ annually. The decrease of burials had amounted to $44\frac{5}{7}$ annually, and the increase of births to $74\frac{2}{7}$ annually. He shows that at the period when he wrote, York rather exceeded the healthiness of any great town of which registers had been published, the deaths being only 1 in $28\frac{1}{4}$, while, in Vienna, they were 1 in $19\frac{1}{2}$, and in London 1 in $20\frac{3}{4}$. Liverpool and Manchester approached nearest to York in point of healthiness; the deaths in the former being 1 in $27\frac{7}{10}$, and the latter 1 in 28.†

It appears, from a paper by Mr. Panton, that the population in the Isle of Anglesey had increased in the year 1773. But his statements are not sufficient to give us any exact data on the subject. He conceives that the increase may be owing to the substitution of potatoes for herrings as an article of food, a notion too vague to merit any serious attention.‡

We have a very exact account of the population of Madeira in the year 1767, by Dr. Thomas Heberden, from an actual survey which he caused to be made, on purpose to ascertain the point. The result was as follows:—in the year 1743 the number of inhabitants amounted to 53,057, in the year 1767 to 64,614. Hence it follows, that the increase is 1,0082 per cent. per annum, and the numbers in the island double every eighty-four years.§

In the year 1738 a curious book was published by Mr. Kersseloom, on the number of people in Holland and West Friesland, as also in Haerlem, Gauda, and the Hague; a particular account of which is given in several papers in the Philosophical Transactions. We shall give the number

---

* Phil. Trans. 1774. Vol. lxiv. p. 67., Vol. lxv. p. 85., and Vol. lxviii. p. 131.

† Phil. Trans. 1782. Vol. lxxii. p. 35.

‡ Phil. Trans. 1773. Vol. lxiii. p. 180.

§ Phil. Trans. 1767. Vol. lvii. p. 461.

of of inhabitants in Holland and West Friesland, and in different cities, according to this author.*

| | Inhabitants. |
|---|---|
| Holland and West Friesland - - - - - | 980,000 |
| Amsterdam - - - - - - - - - - - - | 241,000 |
| Haerlem - - - - - - - - - - - - - | 50,500 |
| Gauda - - - - - - - - - - - - - | 20,000 |
| Hague - - - - - - - - - - - - - - | 41,000 |
| London - - - - - - - - - - - - - | 653,600 |

He is at much pains to prove that the number of inhabitants in Paris, is greater than in London. But his proofs are unsatisfactory, and were triumphantly refuted by Mr. Maitland.† At present the number of inhabitants in London, approaches to double the number of the inhabitants in Paris. In travelling through Britain and France, the state of the towns is no less strikingly different than that of the country. In Britain every town abounds with new houses, and buildings are every where rising in great abundance; in France the houses in every town are old, and hardly such a thing as a new house is any where to be seen. More new houses have been built in London alone within these twelve years than in all France for fifty years back.

Dr. Price, in a curious paper which he published in the Philosophical Transactions for the year 1775, gives strong reasons for believing that there is a prodigious preponderancy in favor of the country above the most healthy cities. In Manchester, for example, the number of deaths is one to twenty-eight; but in the neighbouring country only one in fifty-six dies. He has brought evidence that nearly the same disproportion holds in other cases. From a pretty long table given by Dr. Price, in this paper, it appears that the number of male births to that of female is as 20 to 19 nearly. But, in some measure to counteract this, the chance of life in females is greater than in males.‡ It may be supposed, perhaps, that the risk which females run from parturition, counterbalances this greater chance of life. But, from the observations of Dr. Bland, made at the Westminster Dispensary, it appears, that the deaths from parturition amount only to 1 in 270.§

We shall finish this part of our subject with noticing a curious paper by Dr. Arbuthnot, the celebrated satirical

* Phil. Trans. 1738. Vol. xl. p. 401.

† Phil. Trans. 1738. Vol. xl. p. 407.

‡ Phil. Trans. 1775. Vol. lxv. p. 424.

§ Phil. Trans. 1781. Vol. lxxi. p. 355.

writer writer, and associate of Swift and Pope; in which he demonstrates, that the constant equality kept up between the sexes is an unanswerable argument in favor of the existence of a Divine Providence. For the chances are so many against this equality, that it could not be supposed to take place unless it were so ordered by the will of the Divine Being, for the express purpose of preventing the possibility of the extinction of the human species.*

---

The following observations made to the Committee appointed to examine the laws relative to the Surgeons' Pupils at St. George's Hospital, and to consider of the best method of improving their education, have been thought, by an eminent physician and teacher, who has communicated them to us, too valuable to be lost. Though some years have elapsed since they were written, we do not perceive that medical education has become so meliorated in this metropolis as to supersede their publication.

In moving for the present Committee, we have two principal objects in view: the one is a restoration, as far as may be useful, of the ancient discipline of the hospital, respecting the surgeons and their pupils; the other is, the introducing such improvements for the purposes of instruction as are now, and have been, for some time, adopted by hospitals of the first consequence.

In the first place, therefore, as far as relates to themselves, they leave all such rules now existing, as are not become useless, in *statu quo;* but they are of opinion, that it will be indispensably necessary for the four surgeons belonging to this hospital, to visit all their patients twice a-week, and to be present themselves at the dressing them once, and to visit them on the remaining days of the week, as often as may be necessary.

They further think, that they ought to meet every Friday in consultation, without being summoned; and that any defaulter ought to be reported to the weekly board, and from thence referred to a General Court, to be dealt with for such breach of duty as they shall see proper; the surgeons being perfectly satisfied that such a regulation is of the utmost importance for the good of the patients; and that the close attendance of the surgeons is indispensably necessary for the instruction of the pupils.

They wish also to have it resolved, That no operation shall be performed, but on extraordinary occasions, such as accidents, except on Mondays, Wednesdays, and Fridays;

---

* Phil. Trans. 1710. Vol. xxvii. p. 186.

and and that no summonses shall be sent out for that purpose. If any particular day is assigned for operations, as was formerly the practice, and is common now at other hospitals, the pupils are ready to attend on that day, but frequently discontinue or relax in their attendance on other days, and by that means are ignorant of many essential parts of that knowledge, which can only be attained by a general attendance at an hospital. The pupils, therefore, should be taught, that a sufficient knowledge of their profession cannot be acquired, without a constant and daily attendance.

They hope the Committee will determine, that there shall be two hours allowed for the chirurgical business of the hospital, from eleven o'clock till one. That the dressings shall be finished at a quarter after twelve, and that the time that remains shall be appointed for extra-business, if there be any. By extra-business is understood, the attendance on the board, on operations, on consultations, and the examination of morbid bodies.

They hope the Committee will direct that every surgeon shall examine, or cause to be examined by some other surgeon of the hospital, the morbid bodies, in the presence of all their pupils, of which notice shall be given on the preceding day, unless there be a convenient time for the examination on the immediate day.

We are further of opinion, that it will be proper for the Committee to resolve, that an operation shall be performed on a dead body, attended with explanations, or a lecture be given on some of the principal parts of surgery, once a-week, by one of the surgeons, in rotation, during nine months in the year, and that *gratis*.

We recommend it further to the Committee, and propose it as a matter of great utility, that they will direct, that a book shall be kept by the house-surgeon, for the instruction of the pupils, wherein shall be entered the material cases, the admission of the patients, their treatment, and the event; together with the appearances on the examination of morbid bodies. That each pupil shall be allowed to take a copy of such entries, at his leisure; and that Saturday, in every week, shall be set apart for that purpose.

In the second place, respecting the pupils, we wish that the Committee will resolve, that to whomsoever the pupils pay the fee for attending this hospital, they shall bring certificates of their having been bred up to the profession, and of their good behaviour, as, till of late, has been customary here, and, in some degree, the practice at other hospitals. That they shall be entered at the weekly board by the junior surgeon, and that they be told, that, if they do not comply with the rules enjoined them, they may be reprimanded or expelled, without retaining any part of the fee paid down on entering. This rule ought to be strictly enforced, as it will cause good behaviour and subordination, and prevent ignorant and improper persons from intruding themselves into the profession.

We wish the Committee further to resolve, that no pupil shall be entered for a less period than one year, or half a year, except during the time of war, when it may be necessary for young men, destined for the sea or land service, to get what knowledge they can, and such, as the usual short notice will admit of.

The last regulation, allowing the attendance of quarterly pupils, is of a private nature, and was never sanctioned by the Board. It was first adopted in the time of war, and afterwards improperly continued, as it must be readily admitted, that no material knowledge can be acquired within so short a space of time as that of a quarter of a year.

We are further of opinion, that it will be proper for the Committee to resolve, that every pupil shall be considered as receiving his instructions from the hospital at large, and consequently under the care of all the surgeons alike.

That no pupil shall be allowed to dress the patients, unless he has attended the hospital three months.

That no pupil shall be capable of being chosen the house-surgeon, unless he has been a dresser for six months, and unless he is, at the time of his election, a yearly pupil of the hospital.

That they dress in turn, and according to seniority.

That the time of attending the hospital shall be every day, from the hour of eleven till one.

That they shall be sent for, in case of operations arising from accidents, if they lodge within a reasonable distance.

That no leave of absence shall be granted for a longer time than three months.

That no pupil shall be allowed a certificate but in cases of strict attendance, and that it be applied for at the time of his leaving the hospital, unless he is absent on foreign service.

J. GUNNING.
WM. WALKER.
T. KEATE.

*St. George's Hospital, May 29th,* 1793.

The above statement, drawn up for the information of the Committee, was adopted by them, as their Report to the special General Court, now called for the above purpose; and was by them directed to be printed, and sent to all the Governors.

